# Aspirin challenge and desensitization in patients with suspected AERD in Qatar

**DOI:** 10.5339/qmj.2022.fqac.14

**Published:** 2022-03-28

**Authors:** Sally Khalil, Salma Taha, Maryam Al-Nesf, Hassan Mobayed

**Affiliations:** ^1^Allergy and Immunology Division, Department of Medicine, Hamad Medical Corporation, Doha, Qatar E-mail: SKhalil3@hamad.qa

**Keywords:** AERD, desensitization, NSAIDs

## Abstract

Background: Aspirin-exacerbated respiratory disease (AERD) is a chronic disease characterized by chronic rhinosinusitis, nasal polyposis, asthma, and intolerance to nonsteroidal anti-inflammatory drugs (NSAIDs). Aspirin challenge is considered the gold standard for diagnosing AERD. Many patients with AERD have reported clinical benefits when desensitized to aspirin and maintained on daily aspirin therapy. In this study, we have summarized aspirin challenges and aspirin desensitization in our division during the past ten years.

Methods: We reviewed aspirin challenges and desensitization procedures performed in the Allergy and Immunology Division at the Hamad Medical Corporation, Doha, Qatar, between 2010 and 2020 from our procedures log registry and reported the results of the procedures.

Results: The procedures were performed for patients with chronic rhinosinusitis, nasal polyposis, and bronchial asthma with a historical reaction to NSAIDs or those never exposed to NSAIDs. The challenge and desensitization procedure protocol is outlined in table.1. Of the 45 procedures performed, 36 (80%) patients reacted during aspirin desensitization; and their characteristics, historical reaction to NSAIDs, provoking dose, length of desensitization, and types of reactions were reviewed. Of the reactors, 32 (88%) patients completed aspirin desensitization successfully. The mean ( ± SD) age of patients was 46 ( ± 11.6) years, and 51% were women. The historical symptoms were asthma symptoms (56%) and naso-ocular (21%). The common (71%) reaction during the procedure was asthma symptoms, and 29% had naso-ocular symptoms. The provoking dose was 50–75 mg in most patients. The desensitization procedure was carried out over 2 days in most patients; however, 29% of the patients needed more than 2 days to complete the desensitization. None of the reactors needed emergency epinephrine use or hospital admission.

Conclusion: In our review, desensitization was successful in all the patients who reacted to aspirin, and it was the only therapeutic choice for patients with AERD before the era of biologics. The procedure was well tolerated in most patients. Aspirin challenge was positive in 80% of our patients with suspected AERD, and this has an important diagnostic value that may help in choosing the proper biologic, such as dupilumab, for these patients.

## Figures and Tables

**Table 1:** Hamad Medical Corporation aspirin challenge desensitization protocol

Step	Day	Time point (minutes)	Aspirin dose (mg)	Cumulative aspirin dose (mg)

1	1	0	25	25

2	1	90	50	75

3	1	180	75	150

4	1	270	100	250

5	1	360	observation	250

6	2	0	150	400

7	2	90	300*	700

8	2	180	observation	700


If a reaction occurs, repeat the reaction's step after symptoms are treated and resolved.

*Patients successfully desensitized to aspirin can be treated with ASA 650 mg twice daily for 6 months and be followed accordingly.

